# Morphological, molecular and FTIR spectroscopic analysis during the differentiation of kidney cells from pluripotent stem cells

**DOI:** 10.1186/s40659-017-0119-6

**Published:** 2017-04-04

**Authors:** Monica Maribel Mata-Miranda, Gustavo Jesus Vazquez-Zapien, Marlon Rojas-Lopez, Virginia Sanchez-Monroy, David Guillermo Perez-Ishiwara, Raul Jacobo Delgado-Macuil

**Affiliations:** 1 Centro de Investigación en Biotecnología Aplicada, CIBA-Tlaxcala, Instituto Politécnico Nacional, 90700 Tepetitla, Tlaxcala Mexico; 2 Laboratorio de Biología Celular y Tisular, Escuela Médico Militar, Centro Militar de Ciencias de la Salud, Secretaría de la Defensa Nacional, 11200 Mexico City, Mexico; 3 Laboratorio de Embriología, Escuela Médico Militar, Centro Militar de Ciencias de la Salud, Secretaría de la Defensa Nacional, 11200 Mexico City, Mexico; 4 Escuela Nacional de Medicina y Homeopatía, Instituto Politécnico Nacional, 07320 Mexico City, Mexico

**Keywords:** Pluripotent stem cells, Differentiated kidney cells, Vibrational spectroscopy, Fourier transform infrared, Principal components analysis

## Abstract

**Background:**

Kidney diseases are a global health problem. Currently, over 2 million people require dialysis or transplant which are associated with high morbidity and mortality; therefore, new researches focused on regenerative medicine have been developed, including the use of stem cells.

**Results:**

In this research, we generate differentiated kidney cells (DKCs) from mouse pluripotent stem cells (mPSCs) analyzing their morphological, genetic, phenotypic, and spectroscopic characteristics along differentiation, highlighting that there are no reports of the use of Fourier transform infrared (FTIR) spectroscopy to characterize the directed differentiation of mPSCs to DKCs. The genetic and protein experiments proved the obtention of DKCs that passed through the chronological stages of embryonic kidney development. Regarding vibrational spectroscopy analysis by FTIR, bands related with biomolecules were shown on mPSCs and DKCs spectra, observing distinct differences between cell lineages and maturation stages. The second derivative of DKCs spectra showed changes in the protein bands compared to mPSCs. Finally, the principal components analysis obtained from FTIR spectra allowed to characterize chemical and structurally mPSCs and their differentiation process to DKCs in a rapid and non-invasive way.

**Conclusion:**

Our results indicated that we obtained DKCs from mPSCs, which passed through the chronological stages of embryonic kidney development. Moreover, FTIR spectroscopy resulted in a non-invasive, rapid and precise technic that together with principal component analysis allows to characterize chemical and structurally both kind of cells and also discriminate and determine different stages along the cell differentiation process.

## Background

Kidney diseases are a global public health problem, worldwide mortality rates between 50 and 80%. Currently, the leading cause of end-stage renal failure is diabetes [1, 2]. Over 2 million people now require renal replacement therapy, but this likely represents less than 10% of those who need [3, 4]. When a sufficiently extensive chronic renal injury occurs, whatever the cause, kidney function progressively declines until reaching terminal renal failure with no treatment capable of reversing the process [5].

The kidney performs essential physiological functions such as the excretion of metabolic waste products and, homeostatic and synthesis functions. However, some of these roles are not compensated by current replacement therapy [5, 6].

Available treatments include replacement function by dialysis or transplant, but unfortunately, these therapies are associated with a high morbidity and mortality, and kidney transplantation is limited by the shortage of donor organs, immune rejection and lifelong treatment with immunosuppressive [3].

Besides, despite significant advances in the understanding of renal failure (RF) and the use of alternative therapies, patient’s quality of life tends to decrease, and kidney damage is associated with a significant number of complications. Consequently, because of the increased rate of RF in terminal stage and few alternative treatments, new investigations focused on regenerative medicine have been developed, including the use of stem cells (SCs) [7].

Embryonic or adult SCs by definition are characterized by their self-renewal and potentiality, peculiarities that allow them to give rise to more SCs and differentiate into various cell lineages under appropriate conditions. According to their potentiality, embryonic stem cells (ESCs) are classified as totipotent and pluripotent, both of them can differentiate into the three germ lines. Non-embryonic or adult stem cells are considered multipotent and unipotent, and their potential for differentiation is restricted [7, 8].

ESCs are obtained from inner cell mass, characterized by unlimited self-renewal and pluripotentiality, which allow them to differentiate into various specialized cells types in a morphologically and functionally way, characteristics that make them attractive for developing differentiated kidney cells (DKCs) [9].

The kidney is a complex organ composed of several cell types, and it is conformed approximately by 11,000 nephrons. The essential components of the nephron include renal corpuscle, tubules and interstitial space [10]. The understanding of molecular basis of renal organogenesis is crucial for the development of research that includes ESCs differentiation into DKCs.

More than 300 genes involved in nephrogenesis have been identified [10, 11]; *PAX2* (paired box 2) gene operates at the initiation of kidney organogenesis, contributing to specification of the nephrogenic territory and metanephric mesenchyme (MM) development; Glial derived neurotrophic factor (GDNF), released by metanephric blastema (MB) induces nephric duct epithelial cells to proliferate, giving rise to the ureteric bud and subsequently the collecting ducts. After that, MB mesenchymal cells begin to synthesize and to secrete GDNF after *WT1* (Wilms Tumor 1) gene expression and activation [11, 12].

Many early patterning genes including *WT1*, *PAX2*, *SOX2* (SRY-box 2) and *WNT*-*4* (Wingless-type MMTV Integration Site Family, member 4) are expressed at the beginning of ureteric bud-MM interaction [13], directing the mesenchymal cells to condense around the tips of ureteric bud, to continue growth and branching of the ureteric bud into the MM. This process is followed by the formation of a vesicle that evolves into “comma” and “S” bodies, which will give rise to the glomerulus. As a result of this process, MM differentiates into nephrons, while the ureteric bud branches result in the collecting ducts [13–15].

A great utopia of the regenerative medicine is to replace injured tissues and diseased organs, and the current significant advances in this field are being developed with great speed. In this research, we differentiated mouse pluripotent stem cells (mPSCs) to DKCs, analyzing their morphological, genetic, phenotypic, and biochemical profiles along differentiation by Real-Time PCR, immunofluorescence and vibrational spectroscopy, once there are no experimental works that report the combination of the aforementioned techniques to characterize DKCs from mPSCs, highlighting that vibrational spectroscopy has only been used to characterize the differentiation of ESCs toward cardiomyocyte precursors and pancreatic cells [16–18].

## Methods

### Pluripotent stem cells culture

Mouse pluripotent stem cells (ATCC, VA, USA) were seeded on culture dishes at a density of 50,000 cells cm^2^. To maintain mPSCs in an undifferentiated state, they were co-cultured with mitotically inactive mouse embryonic fibroblasts (MEFs), using mouse ESCs basal medium (ATCC) supplemented with 15% fetal bovine serum (FBS) (ATCC), 0.1 mM 2-mercaptoethanol (Invitrogen, CA, USA) and 1000 U/ml mouse leukemia inhibitory factor (LIF) (EMD Millipore, Darmstadt, Germany); MEFs medium consisted of Dulbecco’s Modified Eagle’s medium (DMEM) (ATCC), supplemented with 15% FBS and 1% penicillin–streptomycin (10,000 IU/ml–10,000 μg/ml) (Invitrogen). MEFs were mitotically inactive using 20 µg/ml mitomycin C (Sigma-Aldrich, MO, USA); culture dishes were incubated at 37 °C in a humidified 5% CO_2_ and 95% air incubator. mPSCs were separated from MEFs monolayer before differentiation and analysis.

### Differentiation of mPSC into kidney cells

To induce mPSCs differentiation to DKCs, we followed the Morizane et al. [19] protocol for differentiating mPSC into renal lineage, making slight modifications. mPSCs were seeded at a density of 50,000 cells cm^2^, using renal differentiation medium containing DMEM with 10% fetal calf serum (FCS) (Promocell, Heidelberg, Germany), 0.1 mM 2-mercaptoethanol, 10 ng/ml activin (R & D Systems, MN, USA), 150 ng/ml GDNF (R & D Systems), 15 ng/ml BMP-7 (R & D Systems), 1 U/ml LIF, 1.25 µg/ml gremlin (R & D Systems), 25 ng/ml growth differentiation factor 11 (GDF11) (R & D Systems) and 10 ng/ml WNT-4 (R & D Systems). DKCs were maintained in renal differentiation medium for 20 days, and the medium was replaced every other day.

### Morphological description

mPSCs and EBs formed by DKCs were sampled at 5, 10, 15 and 20 days of differentiation, to be morphologically analyzed in an inverted microscope (Ti-U Eclipse, Nikon, Japan), determining the EBs diameters, cell shape and confluence. Representative phase contrast microscopy images were obtained. The projected diameter of the EBs was computed by image analysis software (Image-Pro Premier 9.1, MediaCybernetics, MD, USA) assuming a circular cross-sectional area (N = 10 EBs in each day of differentiation).

### RT-qPCR assays

Total RNA of mPSCs and DKCs populations at specific time points of differentiation (days 0, 10, 15 and 20) was isolated with Trizol reagent (Invitrogen) according to the manufacturer’s protocol. Subsequently, cDNA was synthesized using the first strand cDNA synthesis kit (Invitrogen), as per the manufacturers’ instructions. RT-qPCR was performed using an ABI PRISM 7000 Sequence Detection System (Applied Biosystems, CA, USA). Accumulation of PCR products was detected by monitoring the increase in fluorescence, using SYBR Green PCR Master Mix (Applied Biosystems) as the fluorescent reporter.

The relative expression levels were calculated using C_T_ method, which uses the arithmetic formula 2^−ΔΔCT^. Relative RNA levels of all target genes were normalized against the housekeeping gene *β*-*actin.* Primer expresses software for Real-Time PCR ver 3.0 (Applied Biosystems) was used to design the primers for RT-qPCR (Table 1).

**Table 1:** Nucleotide sequences of primer pairs used for real-time qPCR

Gen	Forward 5′–3′	Reverse 5–3
*β*-*actin*	AGAGGGAAATCGTGCGTGAC	AACCGCTCGTTGCCAATAGT
*Oct4*	ACATGTGTAAGCTGCGGCC	TCCAGACTCCACCTCACACG
*SOX2*	AACCGATGCACCGCTACG	TTGACCACAGAGCCCATGG
*PAX2*	CGCTCCAACGGTGAGAAGAG	AGACTCGATCCAGAGCTTCCAG
*WT1*	GCATCCCAGGCAGGAAAGT	TCCTTCCGGCAAACCTGATA
*Ksp*	ACCCAACAGTCAACATCCCTG	GCTTTGTTTTCAGCCCCCTC

### Immunocytochemistry

**Fig. 1:**
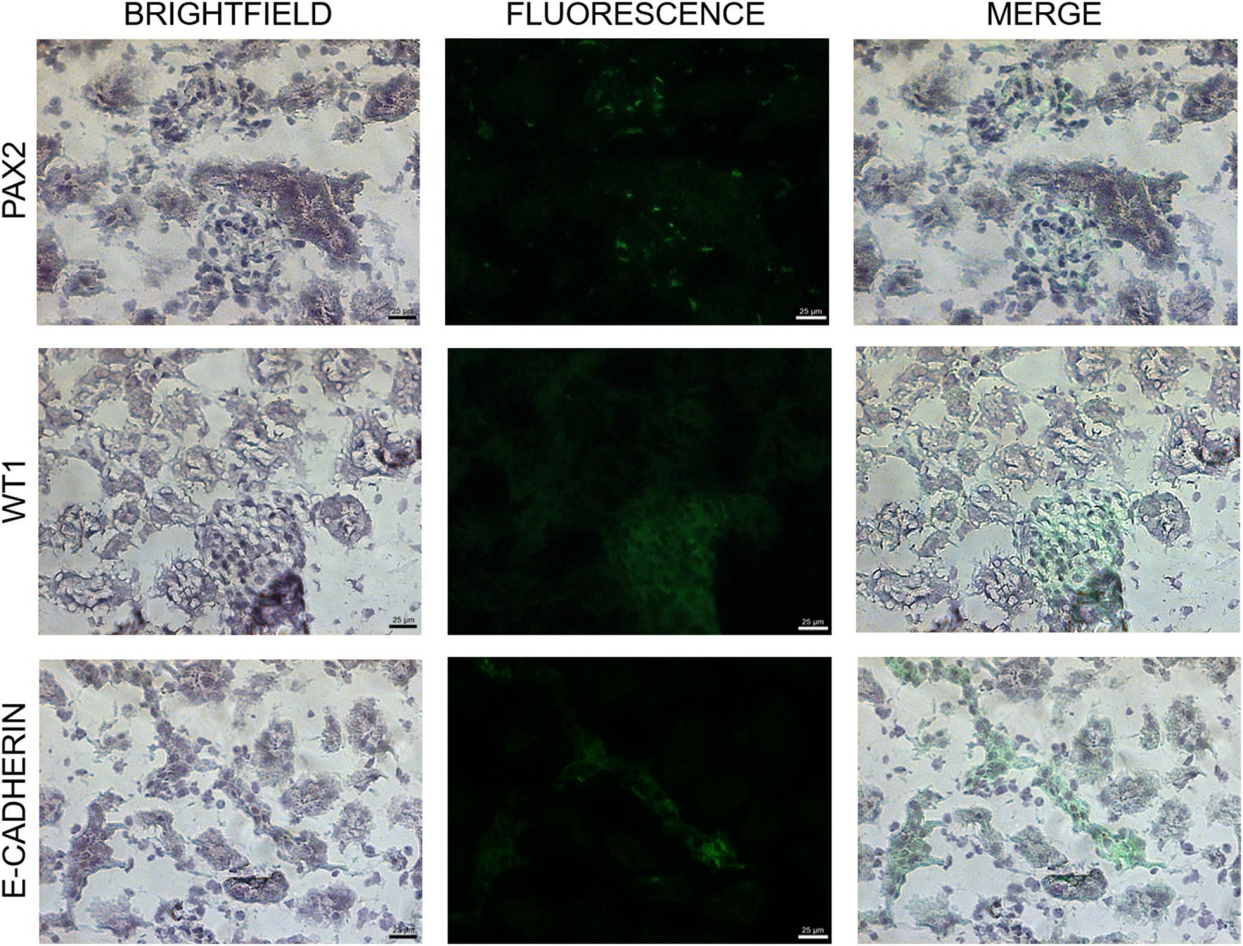
Kidney protein expression using antibodies directed against PAX2, WT1, and E-cadherin proteins on mouse kidney tissue. The samples were counterstained with H&E (positive controls, ×200). The *scale bars* represent 25 μm

mPSCs and DKCs were seeded in chamber slide (Sigma-Aldrich); once mPSCs cultures reached 70% confluence, and DKCs culture fulfilled 18 days of culture, both cell lines were fixed in 4% paraformaldehyde (Sigma-Aldrich) for 30 min, then samples were washed with phosphate buffer solution (PBS) twice. Subsequently, cells membranes were permeabilized using 0.1% Triton X100 (Sigma-Aldrich) in PBS at room temperature for 5 min, after that, samples were washed with PBS and incubated with blocking protein (Dako, Glostrup, Denmark) for 20 min to inhibit nonspecific staining. Immunocytochemical staining was done using rabbit primary antibody anti-Oct4 (1:200, Abcam, Cambridge, UK), mouse primary antibody anti-SSEA1 (1:10, Abcam), rabbit primary antibodies anti-PAX2 (1:10, Santa Cruz, TX, USA), anti-WT1 (1:10, Santa Cruz), and anti-E-cadherin (1:10, Santa Cruz); PSCs antibodies were incubated overnight at 4 °C and kidney antibodies were incubated for 60 min at room temperature. After that, samples were washed with PBS twice, and the conjugated secondary antibodies, Dylight 488 goat anti-rabbit (1:200, Abcam) and Alexa Fluor 647 goat anti-mouse (1:200, Abcam) were incubated for 45 min in darkness. Finally, samples were washed with PBS and coverslipped with 10% glycerol. Microscopic observations were carried out in a fluorescence microscopy (Ti-U Eclipse, Nikon, Japan). Antibodies against PAX2, WT1, and E-cadherin proteins were previously validated on mouse kidney tissue, and the positive controls are shown in Fig. 1.

### Fourier transform infrared spectroscopy

To analyze the structural and biochemical properties of mPSCs and DKCs populations, a Fourier transform infrared (FTIR) spectrometer (Vertex 70, Bruker, Germany) was employed in a wavenumber interval of 4000–400 cm^−1^ (mid-infrared), using the attenuated total reflectance (ATR) sampling mode to measure the infrared absorption of the samples. The instrument has a fixed spectral resolution of 4 cm^−1^. Once mPSCs culture reached confluence, and DKCs fulfilled 10, 15 and 20 days in culture, the cells were trypsinized and centrifuged at 1200 rpm for 3 min, the supernatant was removed, and cells were washed 3 times with PBS, once Santos et al. [20] have stated that cells are preserved for longer periods of time in PBS, getting concentrated samples of ~10^5^ cells in 3 μl which were placed on the surface of the ATR crystal and dried at room temperature for about 15 min to eliminate water excess measuring the spectra until absorption bands related to liquid water were undetectable. The infrared radiation propagated along the crystal to obtain the corresponding spectrum, which was the average of 120 data acquisitions. Three replicates of mPSCs culture and DKCs at different stages of differentiation were analyzed by FTIR spectroscopy.

### Spectral treatment and multivariate analysis

Once all FTIR spectra were acquired (raw spectra), a standard normal variate (SNV) normalization process was applied to them by using UNSCRAMBLER X10.3 CAMO Software, followed by the calculation of their second derivative of each spectrum employing the Savitzky–Golay algorithm, which uses a fitting successive sub-sets of adjacent data points with a small degree polynomial by linear least squares. After the spectra had been treated, they were used as the input information for the application of the principal components analysis (PCA) method, by using the reduction of dimension routine of the IBM SPSS software in both mPSCs and DKCs treated spectra.

### Statistical analysis

Results were expressed as mean ± SD of three separate experiments. One-way analysis of variance (Anova) and Tukey test were used for statistical analysis. A p value <0.05 was considered statistically significant.

## Results

### Morphological description along differentiation process

**Fig. 2:**
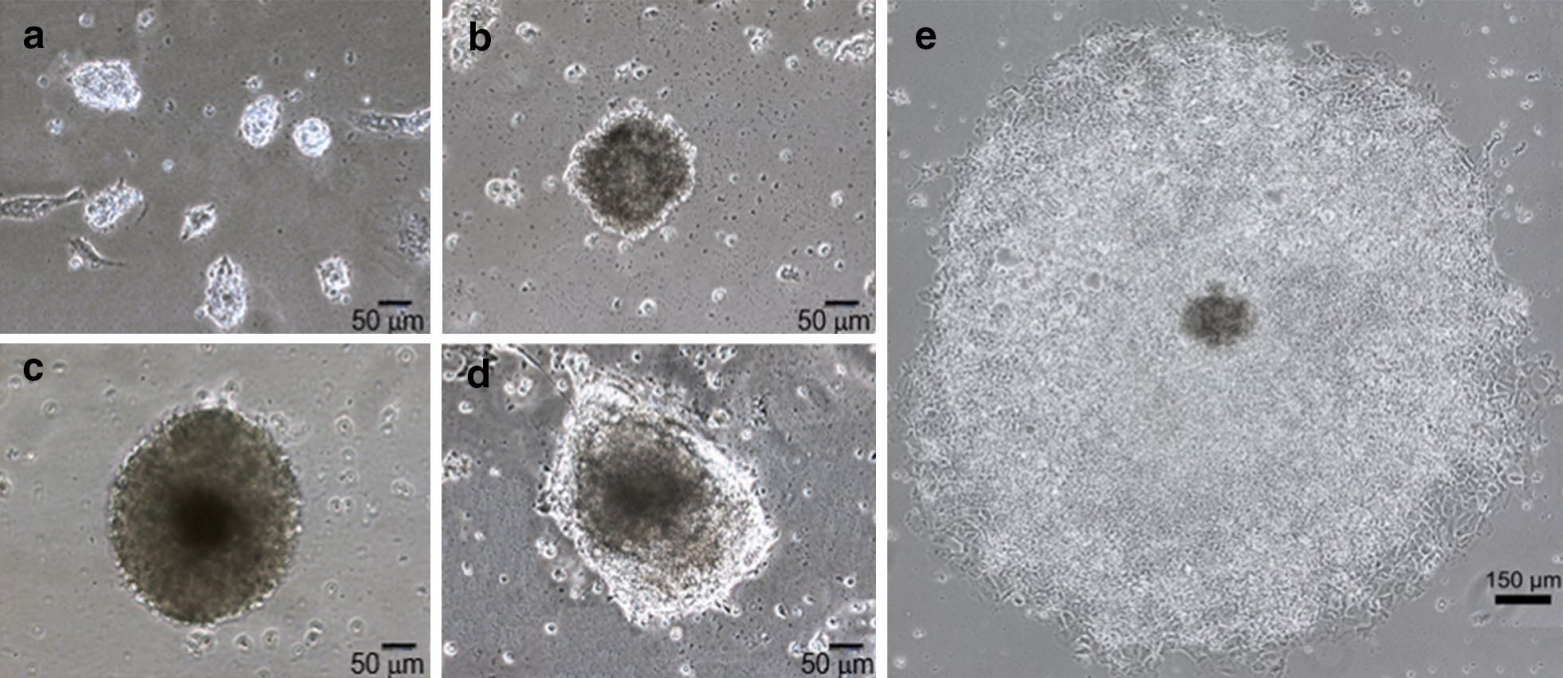
Embryoid bodies (EBs) at different stages of differentiation. **a** Day 5 of differentiation, mouse pluripotent stem cells (mPSCs) on differentiation began to form cell aggregates, called EBs with a diameter of 52.493 ± 0.849 µm. **b** Day 10 of differentiation, cell density of EBs growth, as well as EBs diameters, presenting a diameter of 156.28 ± 11.410 µm. **c**, **d** Day 15 of differentiation, dense EBs were shown with a diameter of 256.934 ± 15.852 µm. **d** The three-dimensionality of the EBs is evidenced. **e** EB on day 20 of differentiation. The EBs on this stage significantly increased in size, presenting a diameter of 1651.39 ± 32.96 µm. A reconstruction by tilling fields was made. Results were expressed as mean ± SD (×100, p < 0.05)

During differentiation process, morphological features of EBs conformed by DKCs were assessed, and representative phase contrast microscopy images (Fig. 2) were obtained at specific time points of differentiation (day 0, 5, 10, 15 and 20). We observed the formation of cell aggregates and their growth according to culture time. At day 5 of differentiation, diameter cell clusters measured 52.493 ± 0.849 µm (Fig. 2a), and cell aggregates tended to form spherical three-dimensional structures formed by typical adherent cells known as EBs. At day 10, EBs diameter measured 156.28 ± 11.410 µm (Fig. 2b). At day 15, an increment of cell density was evidenced, and in the same way, the EBs diameter increased, measuring 256.93 ± 15.852 µm (Fig. 2c); on Fig. 2d, the three-dimensionality of EBs at day 15 is shown. Finally, at day 20 of differentiation, EBs diameter enlarged up to 1651.39 ± 32.96 µm (Fig. 2e). Statistical significance of the EBs diameters was evidenced (p < 0.001).

### Gene expression

**Fig. 3:**
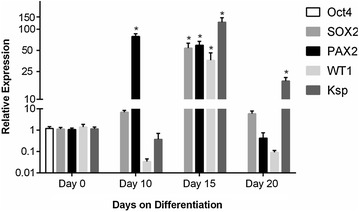
Relative gene expression levels of mouse pluripotent stem cells (*Oct4*, *SOX2*), renal progenitor cells (*PAX2*, *WT1*) and adult kidney cells (*PAX2*, *WT1*, *Ksp*) at different points in time. Triplicate real-time PCR was performed. Expression levels were normalized against *β*-*actin. Bars* represent mean ± SD of N = 3 with 3 biological replicates. *Asterisk* indicate differentiated kidney cells at specific time points of differentiation that showed statistically significant (p < 0.001) in relative expression respect to mouse pluripotent stem cells (day 0 of differentiation)

The expression of pluripotent markers genes (*Oct4* and *SOX2*) and those involved in kidney development (*PAX2*, *WT1*, and *Ksp*), were examined in both mPSCs and DKCs. Changes in relative expression of real-time PCR of the genes mentioned above at specific times of differentiation (day 0, 10, 15 and 20) are summarized in Fig. 3.

The results showed that mPSCs (day 0 of differentiation) expressed *Oct4* and *SOX2* genes, but *Oct4* was undetectable since day 10 of differentiation. On the other hand, the expression of *SOX2* increased gradually until day 15, its expression levels were about 53.64-fold higher than in mPSCs, and at day 20 of differentiation, its expression decreased 5.88-fold. *PAX2* expression (gene involved in marking the nephrogenic territory as well as in the development of MM) increased up to 78.93-fold at day 10, showing after that 0.42-fold decreased at day 20. On the other hand, the expression of *WT1* (gene involved in metanephros initial development, mesenchymal transition in renal epithelium, and expressed in mature podocytes) on day 15 of differentiation increased up to 36.11-fold, but at day 20 its expression was almost undetectable (0.09-fold). Finally, *Ksp* (kidney-specific gene) strengthened its expression until day 15 to 125.82-fold higher than mPSCs, decreasing after that 18.24-fold at day 20 of differentiation.

### Immunofluorescence staining

**Fig. 4:**
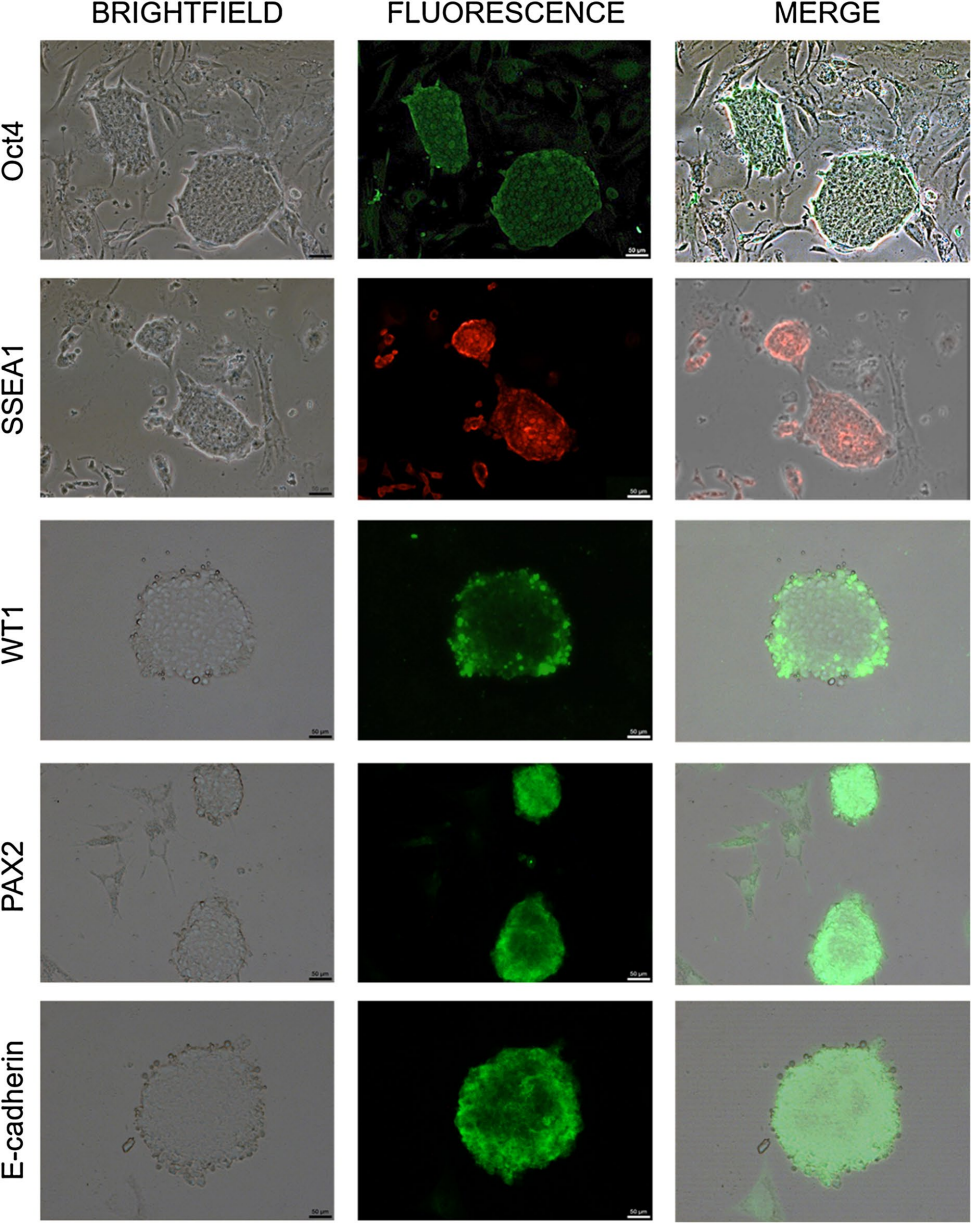
Representative images of immunofluorescence on mouse pluripotent stem cells (mPSCs) and differentiated kidney cells (DKCs). The pluripotency markers SSEA1 and Oct4 were immunodetected on mPSCs cultures, evidencing the undifferentiated state. The immunoexpression of embryonic markers (PAX2 and WT1) and adult kidney markers (PAX2, WT1, and E-cadherin) in the embryoid bodies (EBs) differentiated from mPSCs corroborated the obtention of DKCs (N = 3 with 3 biological replicates, ×100). The *scale bars* represent 50 μm

Protein expression was analyzed by immunofluorescence in mPSCs and DKCs. In agreement with gene expression results, immunofluorescence experiments confirmed the expression of Oct4 and SSEA1 proteins (pluripotent markers) in mPSCs, supporting their pluripotency state. Subsequently, mPSCs were subjected to renal differentiation protocol, and the EBs obtained immunoexpressed PAX2, WT1 and E-cadherin proteins at day 18 of differentiation, corroborating the generation of DKCs (Fig. 4).

### FTIR analysis

**Fig. 5:**
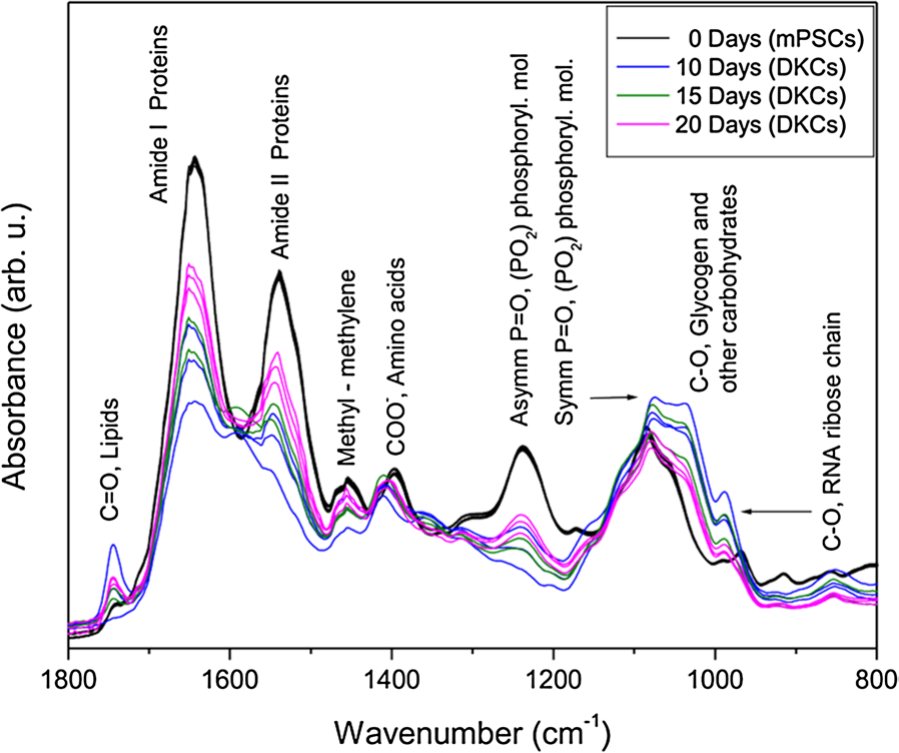
FTIR spectra of mouse pluripotent stem cells (mPSCs) and differentiated kidney cells (DKCs) measured in the fingerprint region (1800–800 cm^−1^). Several absorption bands from lipids, proteins, phosphorylated molecules and nucleic acids are observed (N = 3)

Raw FTIR spectra of both mPSCs and DKCs at specific time points of differentiation were processed by using the SNV normalization, and the resulting spectra are shown in Fig. 5. An absorption band at 1745 cm^−1^ is related to the stretching vibration of C=O ester group of lipids [21], while two intense bands at 1650 and 1540 cm^−1^ are related to amide I and amide II functional groups of proteins respectively [22]. Another band at 1454 cm^−1^ arises from the methyl and methylene groups from lipids and proteins, whereas the band at 1396 cm^−1^ is due to the COO^−^ stretching vibrations of amino acid side chains [21]. The band at 1239 cm^−1^ is related to the P=O asymmetrical stretching of PO_2_ phosphodiester groups from phosphorylated molecules, whereas the band at 1080 cm^−1^ arises from the P=O symmetrical stretching of PO_2_ phosphodiester groups from phosphorylated molecules and glycogen [21, 23]. Other bands at 1154, 1055, and 1015 cm^−1^ respectively are related to C–O vibrations from glycogen and other carbohydrates [20]. Finally, a band around 991 cm^−1^ is mainly due to C–O stretch from RNA ribose chain [17, 23].

**Fig. 6:**
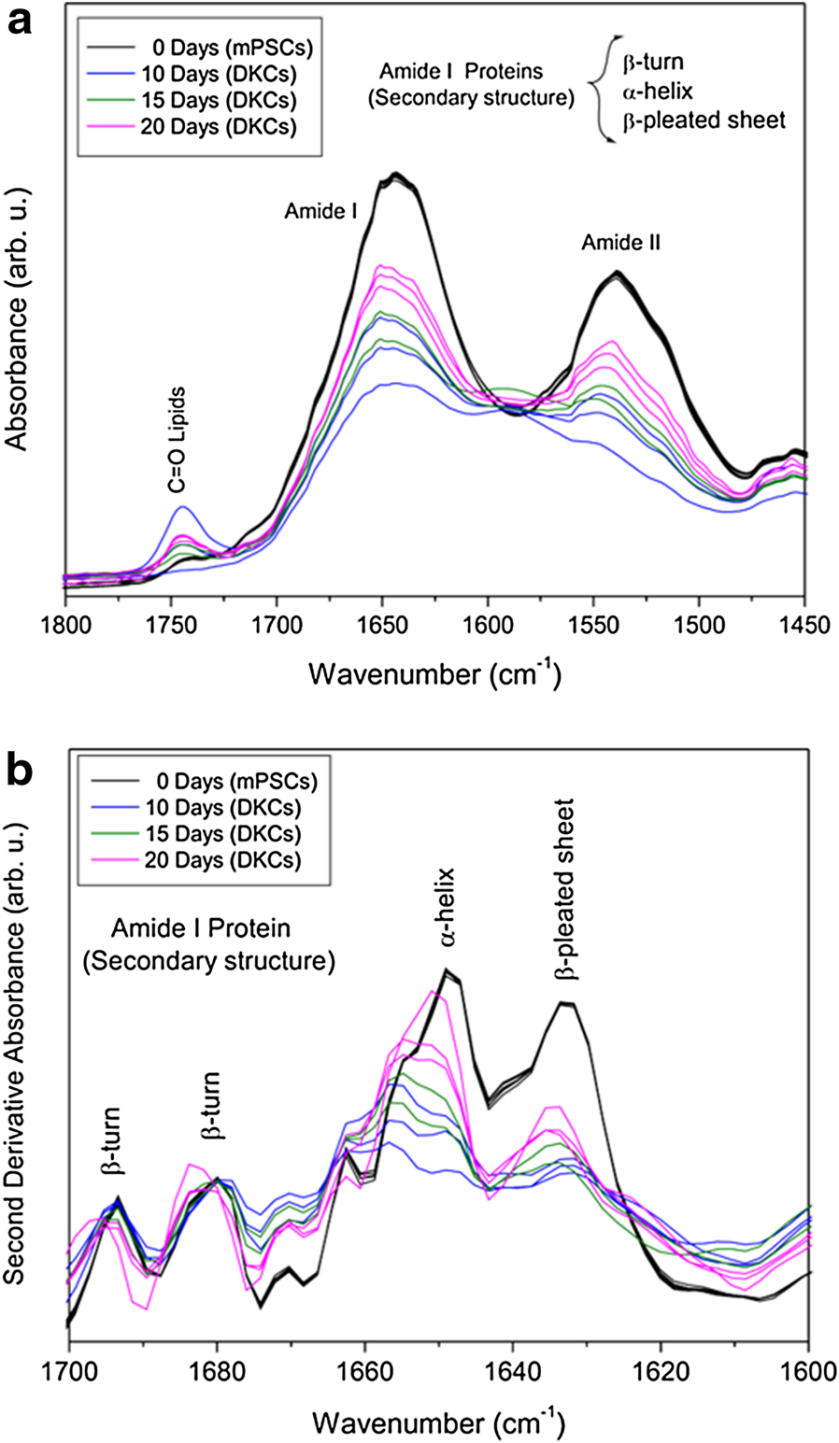
FTIR spectra of mouse pluripotent stem cells (mPSCs) and differentiated kidney cells (DKCs) for several times of differentiation in the proteins region. **a** Raw spectra with SNV normalization, and **b** second derivative spectra (N = 3)

Figure 6a shows the standard normal variate FTIR spectra of both mPSCs and DKCs at specific time points of differentiation in the protein region (1800–1450 cm^−1^), where the amide I (1650 cm^−1^) and amide II (1540 cm^−1^) bands of the proteins are observed. On the other hand, the second derivative spectra of the same samples in the region of the amide I group (1700–1600 cm^−1^) calculated with Savitzky-Golay algorithm allowed the identification of various secondary structures present in the proteins, as β-turns (1694 and 1680 cm^−1^), α-helices (1650 cm^−1^), and β-pleated sheets (1633 cm^−1^) [17, 21–23]; in the same way, differences between mPSCs and DKCs on three bands at 1675, 1670, and 1633 cm^−1^ were observed, which are also related to asparagine, glutamine, and arginine respectively (Fig. 6b) [22].

**Fig. 7:**
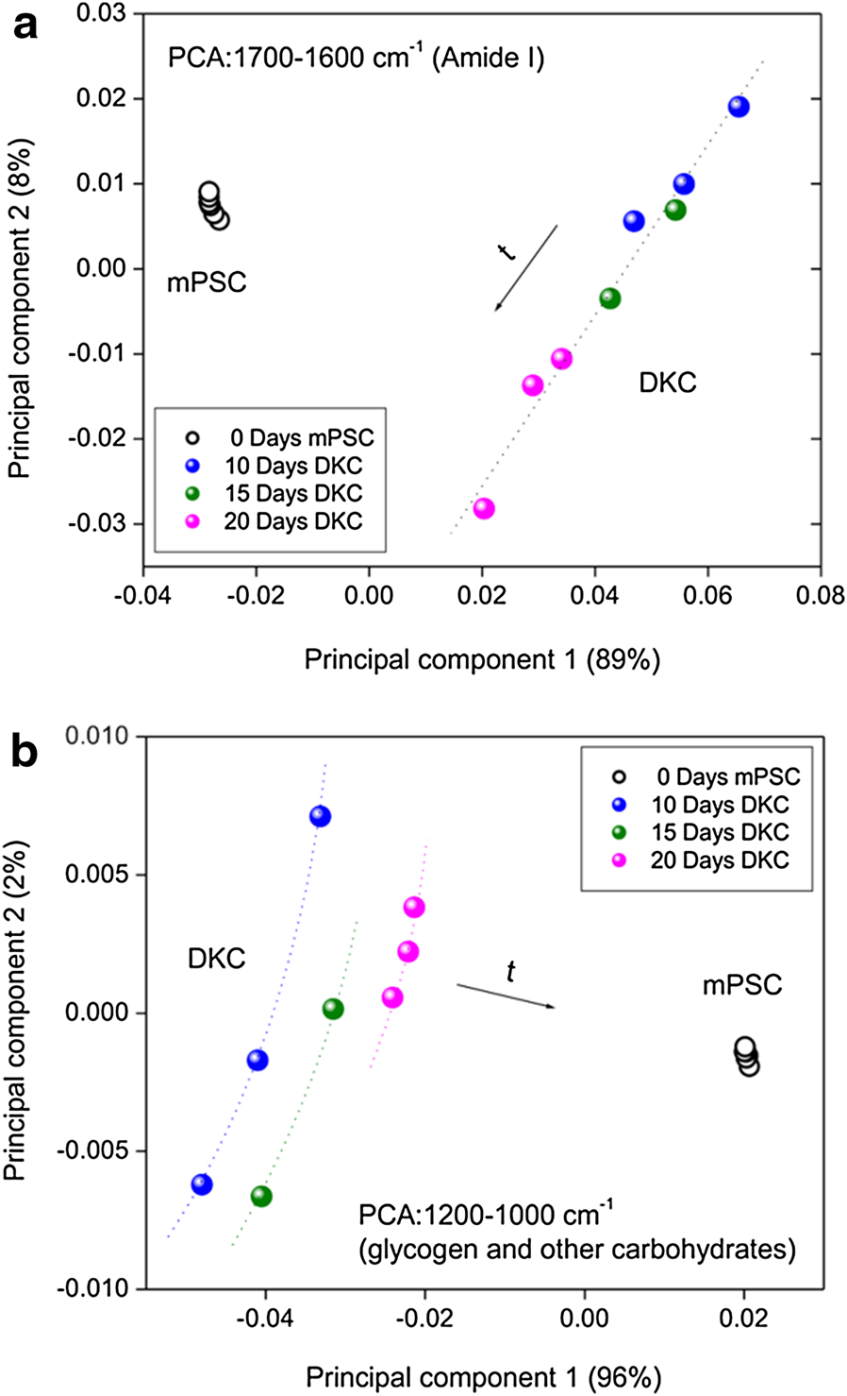
Score plots obtained for the first two principal components after PCA with the second derivative spectra of mouse pluripotent stem cells (mPSCs) and differentiated kidney cells (DKCs) in the following regions: **a** amide I groups from proteins (1700–1600 cm^−1^), and **b** glycogen and other carbohydrates (1200–1000 cm^−1^)

Figure 7 shows the score plots obtained for the first two principal components after PCA with the second derivative spectra of mPSCs and DKCs at 10, 15 and 20 days. PCA was done in two spectral regions: 1700–1600 cm^−1^ (amide I of proteins) and 1200–1000 cm^−1^ (glycogen and other carbohydrates). The first principal component for both explored regions allowed us to discriminated mPSCs from DKCs, and in the region of glycogen and other carbohydrates, the three different stages of differentiation can be distinguished following a radial patron according to the time of differentiation. On the other hand, the second principal component in amide I region evidences the initial and ending differentiation process showing a lineal patron according to the differentiation process.

## Discussion

There is great concern in developing new therapies for RF with the ability to replace the wide range of renal functions [24]. Although it had been considered that the kidney is an organ unable to regenerate, currently, new therapeutic approaches in regenerative medicine have been emerging. This study reports the biochemical characterization of DKCs along differentiation. Previous studies have reported the genetic or phenotypic characterization of DKCs from SCs, but none of them have combined these techniques with FTIR spectroscopy to analyze the differentiation process, and most of published works neither have described the morphological features [3, 9, 19, 34].

During differentiation, three-dimensional EBs were formed, coinciding with some other works that have obtained EBs conformed by specialized cells when ESCs were subjected to a differentiation protocol. It is known that when ESCs are subjected to a spontaneous differentiation in the absence of anti-differentiation factors in vitro, these cells tend to form three-dimensional cell aggregates called EBs [25, 26]. In this sense, it is worth to note that the formation of EBs is a morphological indicator of SCs differentiation.

Once cell expansion and differentiation of mPSCs to DKCs methods were standardized, we proceeded to quantify the relative expression of each transcript (*Oct4*, *SOX2*, *WT*-*1*, *PAX2*, and *Ksp*) at specific time points of differentiation.

First, as expected, pluripotency markers (*Oct4* and *SOX2*) were expressed in mPSC, and according to several authors, during early embryogenesis, *Oct4* and *SOX2* are coexpressed in mouse and human ESCs [27–29]. Furthermore, it has been reported that *Oct4* expression decreases when mESCs are differentiated, until they lose their pluripotency state and the gene is completely suppressed, observing the suppression of *Oct4* at the beginning of the differentiation [30].

Moreover, *SOX2* is an early marker of the inner cell mass, its expression establishes the pluripotent state in mouse blastocyst, and it has been demonstrated that this gene is active during blastocyst formation [31]. So that, in our work, the expression of this gene sustenance the pluripotent state of mPSCs. Nevertheless, we noted that the expression of *SOX2* increased until day 15, and at day 20 this expression decreased, this was probably due to in mice *SOX2* has interference in normal morphogenesis and homeostasis of various tissues, including neural SCs, retinal SCs, taste buds, hair sensory follicles in the ear, and epithelia of trachea, lung, and esophagus [32]. So, several authors have claimed that the expression of this gene is not only limited to PSCs but also adult SCs.

Regarding *PAX2* gene, as mentioned before, it plays an important role in nephrogenesis, remaining expressed along kidney embryonic development, contributing to the specification of the nephrogenic territory and MM development [11]. We have shown that this gene increased its expression at day 10 of differentiation, decreasing thereafter until day 20, results that are similar to those obtained by Imberti et al., who reported that the expression of *PAX2* on renal progenitor cells derived from human induced pluripotent stem cells was observed at day 6 and 12 of culture, and then this expression decreased at day 19 of culture [33]. Furthermore, it is important to mention that the differentiation protocol used in this research enhanced the expression of *PAX2*, once GDNF, BMP-7, and activin were used, which was demonstrated by Morizane et al. [19]; however, Bruce et al. [9] used a different protocol without including the aforementioned factors observing a minor *PAX2* gene expression.

Likewise, it has also been reported that *PAX2* is expressed in kidney epithelial precursor cells and in the proximal tubular epithelial kidney cells when these cells are in culture [34]; in this research, we observed that *PAX2* never stopped expressing even on day 20 of differentiation, probably because after day 15 we had already gotten kidney epithelial cells and tubular epithelial precursor cells, and keeping these cells in culture provoked that this gene maintained expressed.

According to several authors, during normal kidney development *WT1* gene expression is detected at low levels in the MB but increases in the formation of “comma” and “S” bodies upon further differentiation; subsequently, *WT1* expression is downregulated, except in the visceral epithelial cells (podocytes) of the mature glomerulus, where expression can be detected throughout life [35–37]. Our results show that *WT1* gene expression increased at day 15, time corresponding to the formation of “comma” and “S” bodies. After that, the expression decreased at day 20, agreeing with the process of cell maturation, obtaining mature kidney cells (podocytes), highlighting that the expression of *WT1* in our process of differentiation passed through the chronological stages before mentioned. Similarly, our results agree with Imberti et al., who reported the expression of *WT1* at day 0, and its gradual increment at day 6 and 12 [34]; also, consistent with Nishikawa et al. [38] who reported the expression of *WT1* at day 12 with increased expression at day 14, and finally a decrement at day 16.

*Ksp* is exclusively expressed in the kidney; it has been immunolocalized in the basolateral membrane of renal tubular epithelial cells. It has been reported that this gene is firstly detected after the formation of “comma” and “S” bodies, and the expression increases during late gestation and remains high in the adult kidney [39]. In this research, we have shown that *Ksp* expression significantly increased on day 15, probably due to the formation of “comma” and “S” bodies took place; likewise, this gene remained expressed at day 20 of differentiation, which would correspond to the obtainment of adult kidney cells (tubular epithelial cells).

Therefore, the expression of *WT1* and *Ksp* at different stages of differentiation, evidence the obtainment of MB, “comma” and “S” bodies, podocytes and tubular epithelial cells.

In accordance with all the aforementioned, genetic modulation during differentiation process, an unbalance that include genetic downregulation, and overexpression was observed, depending on participation in mature or immature status of cell, however in day 20, genetic expression levels are downregulated with a tendency to basal state suggesting that could be due to the maturation process, conclusion or cellular stability, as has been reported by several authors [30–34, 38, 39].

After RT-qPCR assays, immunofluorescence staining was performed in both mPSCs and DKCs. Some researchers have stated that SSEA1, Oct4, SOX2, and Nanog are typical pluripotency markers [40, 41], reason by which in this research we look for Oct4 and SSEA1 expression on mPSCs (day 0 of differentiation), demonstrating that our mPSCs culture expressed these markers, which confirmed their pluripotency state. After that, as expected, these pluripotency markers were undetectable at day 18 of differentiation on DKCs.

Talking about intermediate mesoderm and MM markers, as mentioned before, it has been reported that PAX2 protein is a marker of intermediate mesoderm, it is expressed in both ductal and mesenchymal components derived from the intermediate mesoderm during the development of pronephros, mesonephros, and metanephros. And although some works have reported the highest expression of this marker on day 6 of differentiation [39], we detected the expression of this protein at day 18, this probably because PAX2 is not just a transcription factor crucial in embryonic kidney development, it is also expressed on collecting ducts of the medulla and cortex as well as the descending portion of the loop of Henle [42]. In the same way, as mentioned in gene expression, we detected a higher PAX2 protein expression in this work than in the Bruce differentiation protocol, due to GDNF, BMP-7, and Activin increase PAX2 and WT1 expression. Furthermore, some other works have reported that WT1 and PAX2 were detected from day 6 to day 12 and then these markers decreased on day 19 [33]. But our results showed that WT1 kept expressed even at day 18 of differentiation; it is worth to note that Imberti et al. [43] got renal progenitor cells, and, as mentioned before WT1 is expressed on podocytes, and it has been used as podocyte marker by many authors.

Otherwise, it is known that E-cadherin is a cell adhesion protein localized at the adherents junction that mediates cell–cell interactions and its expression serves as a marker for epithelial differentiation during the mesenchymal-epithelial transition but, some studies have reported that E-cadherin is predominant expressed in the distal tubule, collecting duct and most medullary segments [3, 44]. Accordingly, the expression of E-cadherin on the EBs obtained in this work at day 18 proves the obtainment of mature kidney cells. These results coincide with those obtained by Ren et al. [3] who detected the expression of E-cadherin in differentiated renal lineages from mESCs by conditioned medium from ureteric bud cells in vitro.

On the other hand, FTIR spectroscopy of mPSCs and DKCs allowed the obtention of information of the main functional groups of the samples (Fig. 5). The obtained spectrum of mPSCs was quite similar to the ESCs spectrum reported by Ami et al. who showed that the most significant spectral changes during ESCs spontaneous differentiation occur in the protein amide (1700–1500 cm^−1^) and carbohydrates and nucleic acid regions (1200–850 cm^−1^) [16], changes that were also detected in this work.

As mentioned before, an increase in the intensity of the C=O band related with C=O esters of lipids for the DKCs at day 10 was shown in comparison with mPSCs; after that, the intensity of this band diminished at 15 and 20 days of differentiation retaining more intense than in mPSCs. About this, it has been reported that phosphatidylcholine synthesis is a metabolic event during compensatory renal growth in kidney development [45], which could be related to the increased intensity of this band along differentiation. In addition, it has been reported that lipid inclusions in the kidney of some mammals are known to be a normal cytoplasmic feature, locating mainly in the pars contorta and the proximal segment of the pars recta of the proximal contorted tubule, being the principal components of the tubular lipids, triglycerides, phosphoglycerides and cholesterol [46]. Besides, it has also been reported that endogenous tissue lipids are the primary source of fatty acids for oxidation and ATP production by the kidney. All those as mentioned earlier, support the importance of the lipids presence in DKCs.

It is known that ESCs core transcriptional circuitries are regulated by phosphorylation, and ESCs differentiation is accompanied by a dynamic modulation of these processes. In this sense Van Hoof et al. have reported that during the induction of the differentiation of hESC, 5222 proteins have been identified, and 1399 of these are phosphorylated on 3067 residues, and have also stated that approximately 50% of these phosphosites are regulated within 1 h of differentiation induction, revealing a complex interplay of phosphorylation networks spanning different signalling pathways and kinase activity, concluding that among the phosphorylated proteins, SOX2 was associated with the pluripotency [47]. Furthermore, Kim et al. have stated that the phosphorylation of Nanog mediated by ERK1 is critical for the down-regulation of Nanog inducing the differentiation of ESCs [48]. In this work, the bands related to phosphorylated molecules were more intense on DKCs, suggesting the phosphorylation and the consequent differentiation of mPSCs.

Additionally, we observed an increase in the intensity of the FTIR spectra in the nucleic acids band, which could be related to changes in RNA content owing to upregulation or downregulation of genes along differentiation process or suggest that the transcriptional switch of the genome started along differentiation [49].

On the other hand, it is known that the amide I and II bands are the two most prominent vibrational bands of the protein backbone, and the most sensitive spectral region to the protein secondary structural components is the amide I band [22]. Considering the aforementioned in this study we calculated the second derivative of this region; and as expected, the secondary structure of the proteins of mPSCs changed when differentiation to DKCs took place, revealing the presence of intense bands related with amide I and amide II functional groups of proteins respectively (Fig. 6a), indicating that different proteins were expressed by the cells during differentiation, agreeing with the results obtained by Ami et al. [16].

In addition to that, second derivative spectra of the amide I group of both mPSCs and DKCs (Fig. 6b) displays several bands related to the secondary structure of proteins as β-turns (1694 and 1680 cm^−1^), α-helices (1650 cm^−1^), and β-pleated sheets (1633 cm^−1^). Comparing these structural spectra, a shift and broadening of the band of α-helices of DKCs with respect to mPSCs was observed. The last probably occurred due to the overlapping with the same bands of the WT1, PAX2, and E-cadherin proteins after differentiation. Furthermore, as mentioned before, the second derivative spectra showed differences between mPSC and DKC on three bands at 1675, 1670, and 1633 cm^−1^ related with asparagine, glutamine, and arginine respectively. About these biomolecules, it is known that the asparagine endopeptidase is an unusually specific endosomal and lysosomal cysteine protease, expressed at high levels in the proximal tubular cells of the mammalian kidney, and it is required for normal kidney physiology and homeostasis [50]. In the same way, the glutamine is the most important donor of NH3 in the kidney playing an important role in acid–base buffering system [51]; oppositely, the protein arginine methyltransferase 4 and 5 have been shown to play essential roles in early embryonic development and in ESCs [52], reason by which we could observe an increment in the intensity of asparagine and glutamine bands in DKCs, as well as an increase of arginine in mPSCs, features that agree with their biochemical cell profiles.

It is important to mention, that mPSCs that were not differentiated into DKCs, did not affect the obtained FTIR results, since the showed results are an average of multiple acquired data, and the non-differentiated cells represent the minority of the obtained cell population.

Similarly to other works, as those one reported by Ami et al. and Cao et al. [16, 21], the FTIR spectra obtained in this research were very complex, but the PCA facilitated us the adequate process to identify the most significant changes along differentiation; for which purpose we used the second derivative spectra of mPSCs and CKDs.

The PCA is a statistical technique that reduces the dimensionality of the variables; it is used in a database with many variables (spectroscopic profiles), reducing the data to a smaller number, losing the least amount of information possible by looking for linear combinations of the original variables. At the same time, the score plots help to understand the correlation structure, forming clusters and trajectories, which allows discriminating different types of cell lineages [18].

The first principal component for both explored regions enabled us to distinguish mPSCs from DKCs easily, evidencing the biochemical and structural changes of the mPSCs once they are subjected to a differentiation process. In the same way, the first principal component of glycogen and other carbohydrates permitted us to distinguish clearly the three different stages of differentiation which can be supported by the second principal component of the amide I, suggesting the expression of various proteins in these stages of differentiation as well as cell complexity (Fig. 7).

In this work, the use of PCA allowed us to discriminate mPSCs from DKCs in a practical way. The application of this methodology could be useful to characterize this kind of cells in very short times, and it is also possible to determine if there are cells that were not differentiated to kidney cells, when the spectroscopic profile of these cells is not content in the specific regions of the PCA for DKCs reported in this work, once the 97 and 99% of the spectroscopic features in the amide I and glycogen and other carbohydrates regions were grouped.

## Conclusions

Regenerative medicine and SCs basic research have opened a new gate for the treatment of kidney diseases. According to this research, we concluded that the induction of mPSCs to DKCs provoked the formation of EBs, which showed growth and maturation directly proportional to the exposure time of differentiation. Regarding gene and protein expression, renal cells were obtained, which passed through the chronological stages of embryonic kidney development. Moreover, FTIR spectroscopy resulted in a non-invasive, rapid and precise technic that together with PCA allows the chemical and structurally characterization of both kinds of cells, also helps to discriminate and determine different stages along the cell differentiation process.

## Abbreviations

RF: renal failure
SCs: stem cells
ESCs: embryonic stem cells
DKCs: differentiated kidney cells
MM: metanephric mesenchyme
MB: metanephric blastema
MEFs: mouse embryonic fibroblasts
FTIR: Fourier transform infrared
PCA: principal component analysis
